# Big data networks: Dynamic Time Warping as a statistical tool for network analysis using Ecological Momentary Assessment data

**DOI:** 10.1192/j.eurpsy.2023.1579

**Published:** 2023-07-19

**Authors:** F. van der Does, W. van Eeden, F. Lamers, B. Penninx, H. Riese, E. Vermetten, K. Wardenaar, N. van der Wee, E. Giltay

**Affiliations:** 1 Psychiatry, Leiden University Medical Center, Leiden; 2 Amsterdam UMC, VUmc, Amsterdam, Netherlands; 3 Psychiatry, Amsterdam UMC, VUmc, Amsterdam; 4 Psychiatry, University Medical Center Groningen, Groningen, Netherlands

## Abstract

**Introduction:**

In recent research, psychological disorders have been increasingly defined as complex dynamic systems in which symptoms are interconnected and influence each other, thereby forming symptom networks. This paradigm shift calls for the analysis and interpretation of relationships between symptoms that are complex, potentially non-linear, and dynamic. Dynamic Time Warping (DTW) is used to measure similarity in temporal sequences, and has recently been found effective in modelling psychopathology symptom networks.

**Objectives:**

We aim to demonstrate that DTW could also be used to model the network structure in Ecological Momentary Assessment (EMA) data.

**Methods:**

355 participants of the Netherlands Study of Depression and Anxiety (NESDA), of which 100 with and 255 without current disorder, completed EMA assessments of 20 symptoms (e.g., feeling sad, tired, satisfied) five times a day for two weeks. DTW analysis was performed on the group level, comparing participants suffering from mood disorders to healthy controls. DTW distances were visualized as an undirected symptom network, in which we adjusted for the average symptom severity per item per person.

**Results:**

DTW analysis of close to half a million symptom scores yielded six symptom dimensions based on their aggregated similarity of changes over time within the participants. Surprisingly, negative affect symptom networks were found to be less strongly connected in those currently suffering from mood disorders than in controls, whereas the network density of (reverse-coded) positive affect symptoms was more closely connected in this group. This is contrary to the results of previous studies, where negative affect-related symptom networks of those with mood disorders were found to be more strongly interconnected.

**Conclusions:**

DTW is a promising new technique for analyzing EMA data and modeling dynamic symptom networks at both the individual and group levels. Using EMA data, symptom networks and dimensions can be modeled with great structural and temporal detail. Incorporating the temporal symptom dynamics may highlight the importance of the independent trajectories of negative mood symptoms.

**Disclosure of Interest:**

None Declared

